# Flow cytometry-based enrichment for cell shape mutants identifies multiple genes that influence *Helicobacter pylori* morphology

**DOI:** 10.1111/mmi.12405

**Published:** 2013-10-16

**Authors:** Laura K Sycuro, Chelsea S Rule, Timothy W Petersen, Timna J Wyckoff, Tate Sessler, Dilip B Nagarkar, Fakhra Khalid, Zachary Pincus, Jacoby Biboy, Waldemar Vollmer, Nina R Salama

**Affiliations:** 1 Division of Human Biology, Fred Hutchinson Cancer Research CenterSeattle, WA, USA; 2 Department of Microbiology, University of Washington School of MedicineSeattle, WA, USA; 3 BD BiosciencesSeattle, WA, USA; 4 Division of Science and Mathematics, University of MinnesotaMorris, MN, USA; 5 Department of Molecular, Cellular and Developmental Biology, Yale UniversityNew Haven, CT, USA; 6 Centre for Bacterial Cell Biology, Institute for Cell and Molecular Biosciences, Newcastle UniversityNewcastle upon Tyne, UK

## Abstract

The helical cell shape of *Helicobacter pylori* is highly conserved and contributes to its ability to swim through and colonize the viscous gastric mucus layer. A multi-faceted peptidoglycan (PG) modification programme involving four recently characterized peptidases and two accessory proteins is essential for maintaining *H. pylori*'s helicity. To expedite identification of additional shape-determining genes, we employed flow cytometry with fluorescence-activated cell sorting (FACS) to enrich a transposon library for bacterial cells with altered light scattering profiles that correlate with perturbed cell morphology. After a single round of sorting, 15% of our clones exhibited a stable cell shape defect, reflecting 37-fold enrichment. Sorted clones with straight rod morphology contained insertions in known PG peptidases, as well as an insertion in *csd6*, which we demonstrated has ld-carboxypeptidase activity and cleaves monomeric tetrapeptides in the PG sacculus, yielding tripeptides. Other mutants had only slight changes in helicity due to insertions in genes encoding MviN/MurJ, a protein possibly involved in initiating PG synthesis, and the hypothetical protein HPG27_782. Our findings demonstrate FACS robustly detects perturbations of bacterial cell shape and identify additional PG peptide modifications associated with helical cell shape in *H. pylori*.

## Introduction

Manual visual screens of transposon mutant libraries for rare bacterial cell shape mutants, though tedious, have been fruitful in uncovering novel machinery that generates cell shape. CreS, the *Caulobacter crescentus* cytoskeletal protein required for that organism's curved rod shape, was first discovered in such a screen (Ausmees *et al.*, 2003), as were Csd1 and Csd4, the peptidoglycan (PG) peptidases we previously reported to be essential for *Helicobacter pylori*'s helical morphology (Sycuro *et al*., 2010; 2012,). One advantage of these types of screens is their indiscriminate nature; any and all types of morphological aberrations may be observed and isolated for study. In our case, two classes of cell shape mutants were discovered in the manual screen: curved rods (*csd1* mutants) and straight rods (*csd4* mutants). However, the unassailable drawback of this methodology is its poor efficiency; hundreds of hours at the microscope are required to saturate these screens and uncover all non-essential genes required for the faithful maintenance of a given organism's morphology.

Fortunately, bioinformatic approaches combined with bacterial genetics and biochemistry have complemented these screens, providing a basic mechanistic understanding of shape generation and the scope of protein machinery involved. In Gram-negative bacteria, cell shape is maintained by a thin peptidoglycan (PG) or murein sacculus which surrounds the cytoplasmic membrane (Typas *et al.*, 2012). PG is made of glycan chains of alternating *N*-acetylglucosamine–*N*-acetylmuramic acid (Glc*N*Ac–Mur*N*Ac) residues that are cross-linked by short peptides. Newly synthesized, uncross-linked pentapeptides have the sequence l-Ala–d-iGlu–*meso*-2,6-diaminopimelic acid (*meso*-Dap)–D-Ala–D-Ala (or Gly). According to current models of PG assembly, high-molecular-weight penicillin-binding proteins (*H. pylori* has one class A PBP, PBP1, and two class B PBPs, PBP2 and PBP3) carry out glycosyltransferase and transpeptidation reactions, the latter catalysing the formation of a tetra–pentapeptide cross-link from two monomeric pentapeptides present on neighbouring glycan strands (see Vollmer *et al.*, 2008; Egan and Vollmer, 2013 for detailed reviews). However, the nascent structure undergoes significant (and likely continuous) remodelling due to the activities of PG hydrolases (Vollmer *et al.*, 2008). Hydrolysis of peptide cross-links as well as trimming of uncross-linked peptides within the *H. pylori* sacculus are required for generation of this organism's characteristic helical shape (Sycuro *et al.*, 2010; 2012,).

As mentioned above, our visual screen uncovered Csd1 as a shape-determining LytM endopeptidase homologue conserved in *H. pylori*. A search for other LytM homologues identified two additional proteins, Csd2 and Csd3, which mutagenesis studies confirmed are also integral for cell shape (Bonis *et al.*, 2010; Sycuro *et al.*, 2010). All three hydrolyse peptide cross-links within the PG sacculus, specifically the *meso*-Dap–d-Ala amide bonds formed by PBPs during PG synthesis. Compared with Δ*csd1* and Δ*csd2* mutants, which are slightly curved to crescent-shaped rods, the morphology of Δ*csd3* mutants is distinct and much more heterogeneous; most cells are highly curved rods that are ‘c'-shaped or concatenations of these curved rods that appear ‘figure-eight’ shaped, although a minority are straight rods (Sycuro *et al.*, 2010). In tune with its unique morphology, Csd3 appears to be the only LytM homologue in *H. pylori* to have a second catalytic activity, that of a dd-carboxypeptidase that trims uncross-linked pentapeptides within the PG sacculus to tetrapeptides (Bonis *et al.*, 2010). Our genetic studies also revealed that disruption of *ccmA*, a gene neighbouring *csd1* and *csd2*, results in curved rod morphology, but this gene's function remains unknown.

Our microscopy-based screen identified two genes whose disruption leads to straight rod morphology: *csd4* and *csd5*. Biochemical studies demonstrated Csd4, an M14 family protease, has dl-carboxypeptidase activity targeting uncross-linked tripeptide to yield dipeptide (Sycuro *et al.*, 2012). Others have shown the Csd4 homologue Pgp1 is required for helical cell shape in *Campylobacter jejuni* (Frirdich *et al.*, 2012). Csd5 sequence and predicted structure did not readily suggest a function, leading us to hypothesize it may play an accessory or scaffolding role in *H. pylori* PG modification.

Double mutants lacking both Csd1/3-mediated cleavage of PG cross-links and Csd4-mediated tripeptide trimming retained the changes in global PG content that were present in each single mutant, suggesting the two types of PG modification independently contribute to *H. pylori*'s helical cell shape (Sycuro *et al.*, 2012). In addition, Δ*csd3csd4* double mutants did not display the ‘c'-shaped morphology of Δ*csd3* mutants, indicating Csd3 is not the only dd-carboxypeptidase capable of generating the tetrapeptide precursor of Csd4's tripeptide substrate (i.e. trimming only occurs in a step-wise manner). Together, these findings suggest a multifaceted PG modification programme determines cell shape in *H. pylori* and that many of the proteins involved still await discovery. At a minimum, we expect *H. pylori* encodes shape-determining proteins that trim uncross-linked pentapeptides and tetrapeptides (dd- and ld-carboxypeptidase activities respectively) upstream of Csd4 tripeptide trimming. However, it is also possible that proteins with dd- and ld-carboxypeptidase activities targeting cross-linked peptides have a bearing on cell shape. Moreover, other dd-endopeptidases that hydrolyse tetra–tetrapeptide or tetra–tripeptide cross-links may work alongside the Csd1–3 LytM homologues to allow bends and twists in the *H. pylori* sacculus.

In order to fill these gaps in *H. pylori*'s PG modification pathway and gain a better understanding of how the modifications work in concert to produce a helical cell body, we explored fluorescence-activated cell sorting (FACS) as a novel method of enriching a transposon mutant library for rare cell shape mutants. Although flow cytometry has been used routinely in eukaryotic cell biology since the 1970s, advancements in detector sensitivity and cell sorting capabilities have recently made this technology applicable to much smaller cells, including bacteria and other microorganisms. Most studies with microbial cells have employed fluorescent biomolecule labelling techniques to survey for viability, gene expression, physiological state, biochemical function or rRNA sequence content (Winson and Davey, 2000; Czechowska *et al.*, 2008). In addition, since light scattering in the forward direction correlates with bacterial cell size and shape (Davey and Kell, 1996), a few investigators have utilized flow cytometry to quantitatively show differences among bacterial populations with distinct morphologies (Milner *et al.*, 1993; Meberg *et al.*, 2004; Gancz *et al.*, 2008). Most recently, Laubacher *et al*. utilized FACS to enrich *Escherichia coli* cultures for rod-shaped cells containing mutations that suppressed highly aberrant branching defects present in the parent (mutant) population (Laubacher *et al.*, 2013).

Here we demonstrate that the differential light scattering properties of our three classes of *H. pylori* cell shape mutants is sufficient to rapidly isolate cells with similar morphologies from complex populations. After a single round of FACS enrichment for cells with lower forward scatter (curvature) than wild-type, we successfully isolated a rod-shaped mutant that contained an insertion in HPG27_477. Because of its profound effect on cell shape, we named this gene *csd6*. Analysis of the PG content of mutant sacculi and incubation of purified protein with isolated sacculi revealed that Csd6 encodes the ld-carboxypeptidase responsible for trimming uncross-linked tetrapeptides in the PG sacculus. Studies of the same FACS-enriched population also led to the identification of two new mutants with more subtle morphological defects. Our findings demonstrate that flow cytometry-based morphological enrichment greatly enhances the efficiency of obtaining rare cell shape mutants from a transposon mutant library, without sacrificing diversity or nuance in the morphologies of the isolated clones.

## Results

### Distinct patterns of light scatter allow morphologically diverse populations of *H. pylori* cell shape mutants to be enriched using FACS

**Fig 1:**
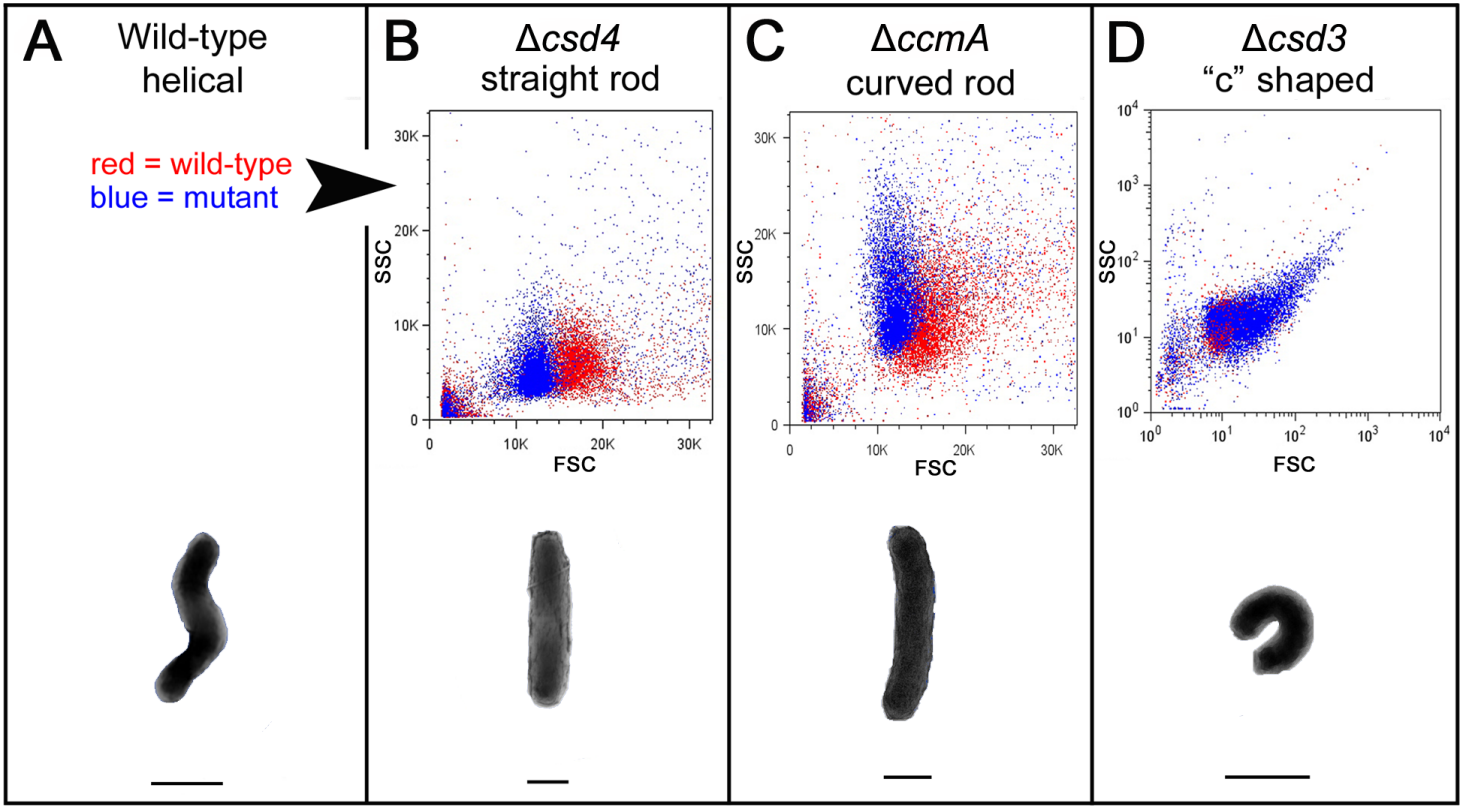
Flow cytometry distinguishes wild-type *H. pylori* cells from three classes of cell shape mutants. B–D. (Upper) Scatter plots of each mutant (blue) are overlaid with wild-type plots (red) generated in the same experiment for comparison; however, not all mutants were analysed in the same experiment. Forward scatter is shown on the *x*-axis (FSC) and side scatter on the *y*-axis (SSC). Note that the axes in (D) are displayed in log scale to show the full range of spectral properties observed with the *csd3* mutant population. A–D. (Lower) Illustration of the characteristic morphology of each strain/shape class. Lines drawn below each cell indicate its bulk width, which may contribute to the observed differences in forward scatter. Images of cells in the schematic are not to scale. Strains used: NSH57, LSH18, KGH10, LSH112.

To examine the feasibility of using FACS to sort *H. pylori* cells according to morphology, we first characterized the light scattering properties of wild-type *H. pylori* and genetically defined mutant populations representing the three known *H. pylori* shape classes: straight rods (Δ*csd4*), curved rods (Δ*ccmA*), and variable/‘c'-shaped rods (Δ*csd3*) (Sycuro *et al.*, 2010; 2012,). Flow cytometry and cell sorting were performed using a BD Influx cell sorter optimized for the detection of small cells (see *Experimental procedures* and Petersen *et al.*, 2012 for further detail). Suspensions of *H. pylori* cells were taken from freshly passaged blood agar plates and cultured in liquid growth media for approximately one doubling time to achieve uniform morphology. To account for slight day-to-day variability in cell preparations and flow cytometer performance, which resulted in variable side scatter (SSC) values (compare wild-type populations in Fig. 1B and C), mutant and wild-type cells were always analysed side by side after being prepared in the same growth media under identical conditions. Using these methods we consistently observed the straight rod Δ*csd4* mutant and the curved rod Δ*ccmA* mutant populations displaying lower forward scatter (FSC) than wild-type, although the Δ*ccmA* mutant population overlapped significantly with wild-type (Fig. 1A–C). In concert with their variable morphology, the Δ*csd3* mutant population displayed a wide range of FSC and SSC values, with most cells exhibiting much higher FSC than wild-type and a correlation of high SSC with high FSC (Fig. 1D). In sum, each of our shape mutants displayed light scattering profiles that were clearly distinguishable from that of wild-type. FSC is roughly correlated with cell size, and we accordingly observed that the loss of curvature and twist, which reduces the bulk width of the cells (see schematic illustrations in Fig. 1), resulted in lower FSC. Conversely, ‘c'-shaped cells have an increased bulk width and correspondingly higher FSC. While normally associated with cell complexity or granularity, SSC has also been speculated to correlate with the absorbance of the bacterial cell wall or possibly cell protein content (Gant *et al.*, 1993). Whether the Δ*csd3* mutant has a specific cell wall defect or difference in cellular content contributing to its high SSC values has yet to be determined.

We next explored the possibility of exploiting these divergent light scattering profiles to increase the relative abundance of cell shape mutants in morphologically complex mixed cultures using FACS. For each of the three cell shape mutants tested with flow cytometry, mixed cultures were prepared with mutant and wild-type strains at ratios of approximately 1:1 and 1:1000 (mut : WT). Based on the scatter plots generated with each mutant and wild-type strain individually, inclusion gates were drawn to capture approximately 10-fold more mutant cells than wild-type and to exclude as much of the wild-type population as possible (exclusion of > 99% of wild-type) (Fig. S1). After sorting each mixed culture with the desired gate, aliquots of the sorted and unsorted populations were plated to selective media to enumerate mutant and wild-type cells. As shown in Table 1, a typical FACS experiment produced > 10-fold enrichment of the shape mutant in a single sort, with > 100-fold enrichment not uncommon.

**Table 1:** FACS enriches for *H. pylori* cell shape mutants from mixed cultures of mutant and wild-type

		Starting ratio ∼ 1:1 mutant : WT			Starting ratio ∼ 1:1000 mutant : WT		
Strains	Shapes	Starting ratio (mut : WT)	Sorted population (mut : WT)^a^	Fold enrichment^b^	Starting ratio (mut : WT)	Sorted population (mut : WT)	Fold enrichment^a^
WT + Δ*csd4*	Helical + straight	0.97	131.00	135.1	0.00097	0.15	154.6
WT + Δ*ccmA*	Helical + curved	2.30	510.00	221.7	0.00230	0.03	13.0
WT + Δ*csd3*	Helical + var/‘c’	0.30	9.90	33.0	0.00030	0.11	366.7

a Underlined values indicate only mutant was recovered from these populations; wild-type valued as our lowest level of detection for ratio calculation. Mixtures of Δ*csd4*, Δ*ccmA* or Δ*csd3* and wild-type were sorted using gates 1, 2 and 3, respectively, as depicted in Fig. S1. Strains used: NSH57, LSH18, KGH10, LSH112.

b Fold enrichment is calculated as the sorted ratio divided by the starting ratio.

### Enrichment for cells with low FSC in an *H. pylori* transposon mutant library yields clones with insertions in *csd4*, *csd5,* and an uncharacterized cell shape locus

Having determined that FACS can be used to boost the relative abundance of non-helical cells that represent as little as 0.1% of the population to upwards of 10% of the population, we employed FACS to enrich our 10 000-clone *H. pylori* transposon mutant library (Baldwin *et al.*, 2007) for clones with cell shape defects. Beginning with a population of approximately 25 000 clones plated from this library, we sorted 250 000 cells using a low FSC gate similar to that shown to enrich for straight to curved rod mutant cells in our feasibility testing experiments (Fig. S2). We collected a 2000 cell gated output, ∼ 600 of which grew up on blood agar; these colonies were pooled and frozen for future study.

To determine the proportion of the sorted population displaying cell shape defects, a total of 290 clones were screened by light microscopy. Approximately 50% of the clones displayed straight rod morphology in the initial visual screens, but the majority of these clones became morphologically unstable, with increasing numbers of helical cells appearing during continued culture in liquid broth or on blood agar. As these clones retained chloramphenicol resistance and thus presumably contained a stable insertion, the cause of the morphological instability is not known. We ultimately identified 43 rod-shaped clones (15% of those visually screened) with stable shape phenotypes (Table 2). Most of these clones contained insertions in *csd4* (84%) and *csd5* (14%), but one clone's insertion did not map to any of the known shape loci, suggesting it may disrupt a previously uncharacterized locus involved in helical shape determination. Although we did not identify any clones with curved rod or variable/‘c'-shaped morphologies in our screening, PCR amplification using transposon-specific primers and primers flanking the genetic loci encoding Csd2/Csd1/CcmA (one operon) and Csd3 yielded numerous products when applied to genomic DNA extracted from the pooled FACS output (Fig. S3). This finding indicates multiple clones with unique insertion sites in these loci are present in the FACS output, but whether the curved rod shape classes were in fact enriched by our low FSC sort is a subject of ongoing investigation. What we have shown here is that a single round of cell sorting for cells displaying low FSC was sufficient to raise the abundance of straight rod shape mutants in the transposon mutant library from ∼ 0.4% [determined in our initial visual screen of the parent transposon mutant library in *H. pylori* strain G27 (Sycuro *et al.*, 2010)] to ∼ 15%, constituting a 37-fold enrichment.

**Table 2:** Prevalence of transposon mutants in the low FSC FACS output with stable straight rod morphology and their disrupted loci

Plating number	Clones screened	Stable rod-shaped mutants	Mutants with *csd4* insertions	Mutants with *csd5* insertions	Mutants with insertions in uncharacterized genes
	*n*	*n* (%)	*n* (%)	*n* (%)	*n* (%)
1	70	14 (20%)	10 (71%)	3 (22%)	1 (7%)
2	96	16 (17%)	14 (87%)	2 (13%)	0
3	124	13 (11%)	12 (92%)	1 (8%)	0
Total	290	43 (15%)	36 (84%)	6 (14%)	1 (2%)

### Disruption of HPG27_477, a putative ld-carboxypeptidase, yields cells with uniform straight rod morphology

As mentioned above, one FACS sorted clone with straight rod morphology did not contain an insertion in any of the genetic loci known to be involved in determining *H. pylori*'s helical cell shape (*csd2*/*csd1*/*ccmA*, *csd3*, *csd4* or *csd5*). Sequencing of this clone's transposon insertion junction revealed an insertion at position +8 of the 993 bp gene HPG27_477, annotated as a hypothetical secreted protein and recently suggested to alter flagellar glycosylation (Asakura *et al.*, 2010). This gene appears to be conserved in all sequenced *H. pylori* strains and in other Epsilonproteobacteria, where it is sometimes annotated as ErfK/YbiS/YcfS/YnhG. This protein family has been renamed YkuD (Pfam PF03734), which refers to a conserved Cys/His-containing catalytic domain found in proteins that catalyse an alternative pathway for PG peptide cross-linking (ld-transpeptidation), the covalent linkage of PG peptides to Braun's lipoprotein (Lpp) in the outer membrane, or possibly PG peptide hydrolysis (Biarrotte-Sorin *et al.*, 2006; Magnet *et al.*, 2007a,b). Protein structure prediction using Phyre^2^ (Kelley and Sternberg, 2009) revealed high confidence alignments of HPG27_477 to ld-transpeptidase structures from *Enterococcus faecium* (PDB 2HKL) and *Mycobacterium tuberculosis* (PDB 3U1Q) (data not shown). However, the absence of both ld-cross-linked muropeptides and Lpp linkages in the *H. pylori* PG sacculus (Costa *et al.*, 1999) suggested HPG27_477 likely does not catalyse the formation of these types of amide linkages, but instead may be a hydrolytic ld-peptidase acting alongside the Csd dd- and dl-peptidases to generate helical cell shape. We thus named HPG27_477 *csd6*.

**Fig 2:**
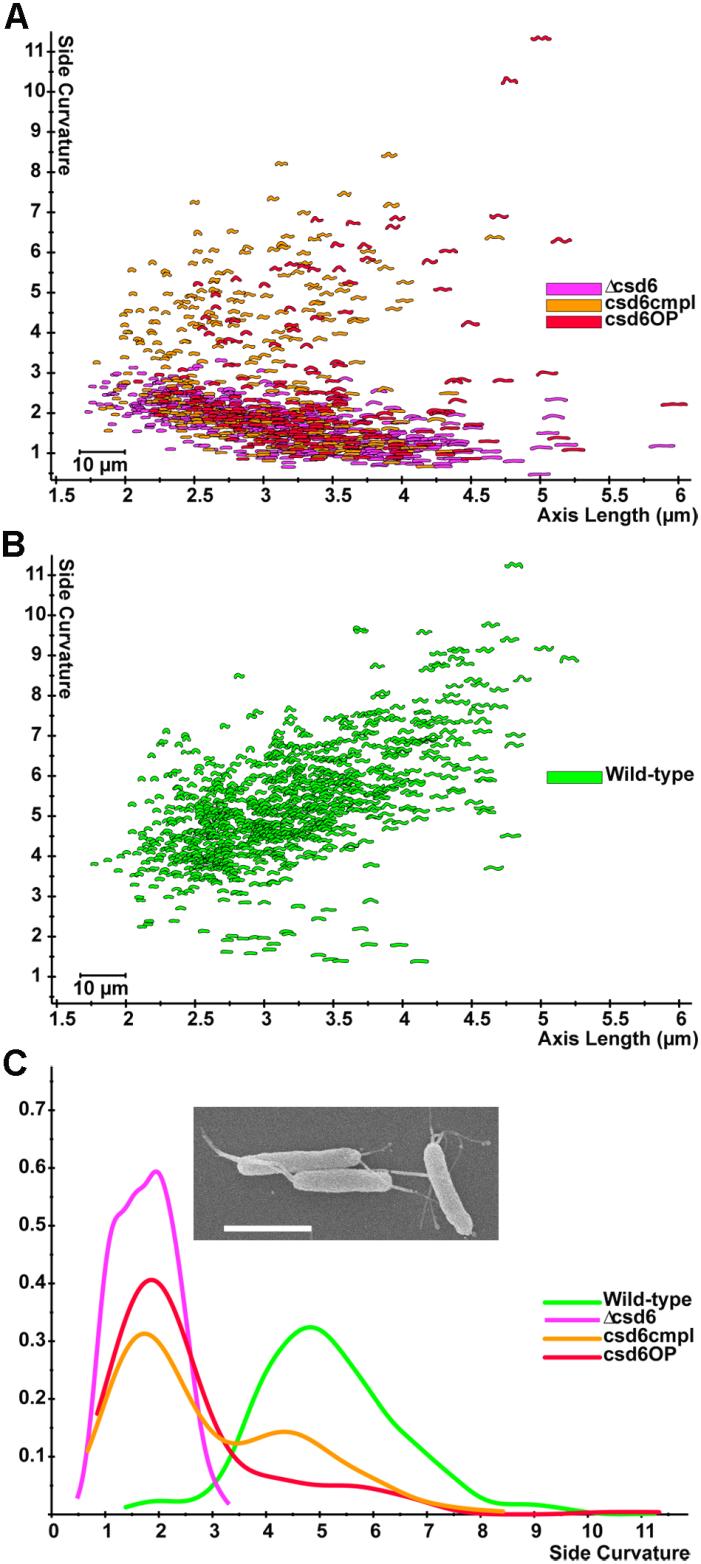
Loss or overexpression of *csd6* perturbs cell shape.A and B. Scatter plot arraying cell length (*x*-axis, μm) and cell curvature (*y*-axis, arbitrary units). Each point depicts the outline of the cell image obtained by phase-contrast microscopy. C. Smooth histograms (kernel density estimate) displaying population cell curvature (*x*-axis, arbitrary units) as a density function (*y*-axis); inset SEM image of *csd6* mutant. Scale bar = 2 μm. *csd6* mutant (Δ*csd6*), complemented (*csd6*cmpl) and merodiploid (*csd6*OP). Strains used: LSH100, TSH17, TSH31, TSH35.

Targeted deletion of *csd6* recapitulated the loss of curvature and straight rod morphology of the transposon mutant clone, confirming its role in cell shape determination. This was quantified using CellTool software to measure the side curvature (a measure of total curvature of the bacterial outline excluding the poles) of populations of cells imaged by phase-contrast microscopy (Fig. 2A and B). However, attempts to complement the mutation by expressing the gene at the *rdxA* locus, as we have done for all previously identified *csd* genes, failed to restore helical shape (data not shown). To test the possibility that expression from the *rdxA* promoter resulted in inappropriate expression levels, we created an expression construct that fused the predicted promoter of the *csd6* containing operon (upstream of HPG27_473) with the *csd6* open reading frame, adding flanking sequences that would direct recombination at an intergenic locus (Langford *et al.*, 2006). When this construct was recombined into the Δ*csd6* strain, we observed a mixed population of normal helical rods and straight rods (Fig. 2A and B). Integration of the *csd6* complementation cassette into a wild-type strain to produce a merodiploid strain again resulted in a mixed population of helical rods and straight rods, but the proportion of straight rods was higher than observed in the complemented strain (Fig. 2A and B). We observed the same heterogeneous shape phenotype for multiple independent transformation clones obtained during the construction of these strains. These findings suggest that either absence or overabundance of Csd6 disrupts helical cell shape generation, resulting in straight rod morphology.

### FACS identifies mutants with altered helical morphologies

**Fig 3:**
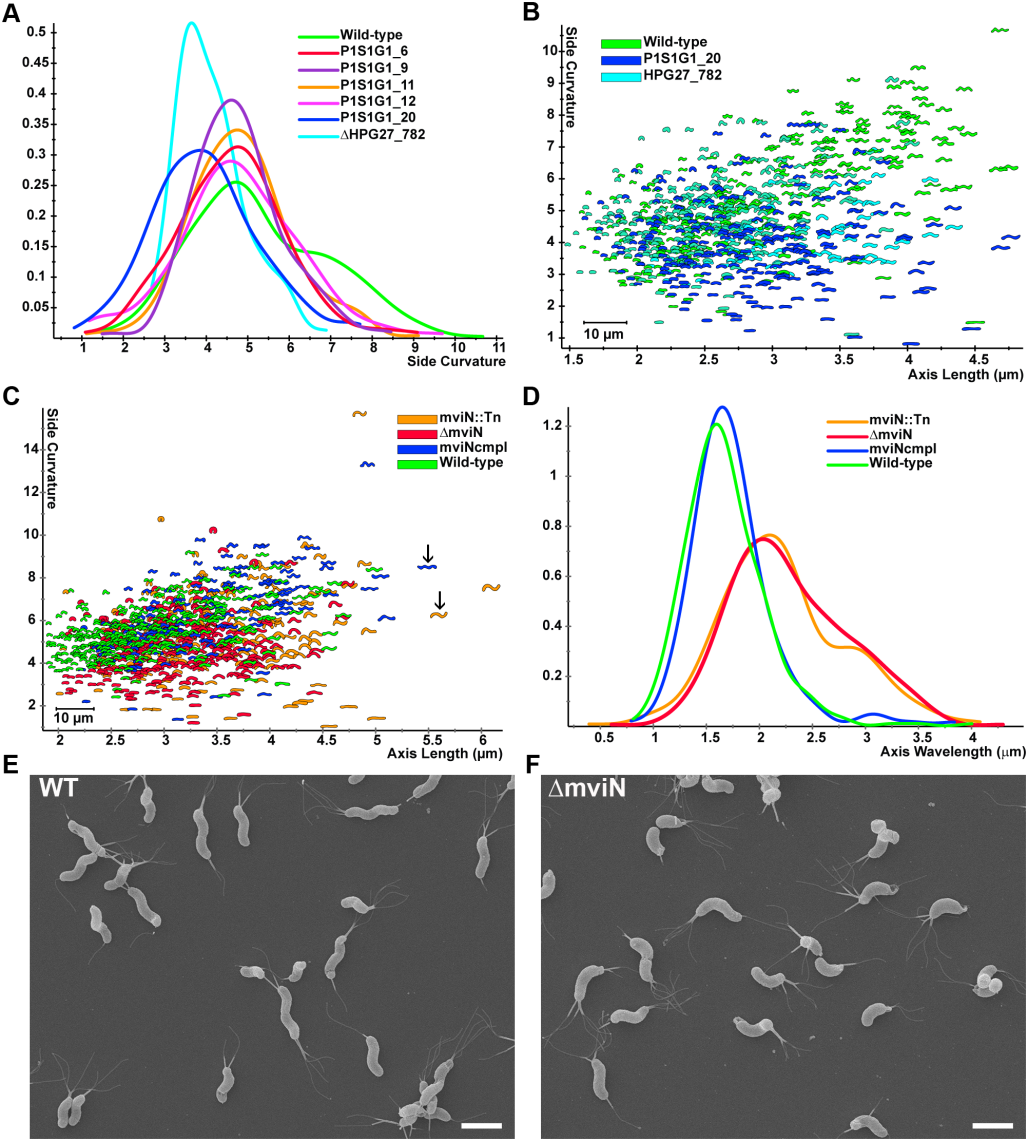
HPG27_782 and *mviN* mutants show subtle morphological changes. A. Smooth histograms displaying population cell curvature (*x*-axis, arbitrary units) as a density function (*y*-axis) for wild-type, selected clones from the low FSC FACS sorted population and HPG27_782 null allele. B and C. Scatter plots arraying cell length (*x*-axis, μm) and cell curvature (*y*-axis, arbitrary units). Each point represents the outline of the cell image obtained by phase-contrast microscopy. B. Comparison of wild-type to HPG27_782 transposon or null mutant strains. C. Comparison of wild-type to *mviN* transposon, null and complemented mutant strains. Arrows indicate *mviN* mutant and complemented cells with similar axis length but different wavelength. D. Smooth histograms displaying population axis wavelength (*x*-axis, μm) as a density function (*y*-axis). E and F. Scanning electron microscope images of wild-type and *mviN* mutant bacteria. Scale bar = 2 μm. Strains used: NSH57, P1S1G1_6, P1S1G1_9, P1S1G1_11, P1S1G1_12, P1S1G1_20, MHH17, LSH100, P4S1G1_29, TSH1, TSH13.

As mentioned above, many clones in the FACS output population displayed helical morphology when subsequently evaluated by light microscopy, but some seemed to show alterations in their helical shape compared with wild-type. From the 70 clones analysed in the first plating of the FACS output population, we saved five clones displaying these more subtle shape phenotypes, in addition to the 14 clones with stable straight rod shape mapping to *csd4*, *csd5* and *csd6* described above and in Table 2. To quantitatively probe for differences in cell shape, we utilized CellTool to examine cell curvature along the long axis of the cell (non-pole regions) across a population of > 200 cells for each of the five clones (Fig. 3A). While four of the clones showed similar side curvature profiles to wild-type, the side curvature profile of P1S1G1_20 showed a side curvature peak that was significantly lower than wild-type (bootstrapped Kolmogorov–Smirnov statistic of side curvature distributions for P1S1G1_20 versus wild-type *P* < 0.00001). The transposon insertion of this clone mapped to +289 of the 345 bp gene HPG27_782, a hypothetical protein conserved among *H. pylori* strains. Targeted deletion of HPG27_782 recapitulated the shift towards lower side curvature in the population distribution, although many cells retained helical shapes (Fig. 3A and B).

We then performed a fourth plating of the FACS output population to specifically look for mutants with other more subtle morphological aberrations. Of 80 clones analysed, one stood out as appearing to have more of an ‘s’ shape compared with wild-type. The transposon insertion of this clone mapped to position +221 of the 1461 bp gene HPG27_838. This gene is annotated as *mviN*. The *E. coli* homologue of this gene has been renamed *murJ* for its postulated role in PG synthesis (Ruiz, 2008). Targeted deletion of *mviN* showed the same altered shape as the transposon insertion (Fig. 3C and D). While a subset of the Δ*mviN* mutant population shows decreased side curvature that could account for the enrichment of this clone in the FACS sort, wild-type and Δ*mviN* mutant populations show considerable overlap in this shape parameter. In contrast, a measure of ‘axis wavelength’ (the average peak-to-peak distance between consecutive helical turns measured along the cell centreline) robustly segregates the wild-type and complemented *mviN* cells from the transposon and deletion *mviN* mutant cells that have significantly longer wavelengths (see Fig. 3C arrows and 3D). Scanning electron microscopy (Fig. 3E and F) confirmed the Δ*mviN* mutants appear to form helical turns with reduced periodicity, consistent with a longer helical pitch. Thus, in addition to a substantial enrichment for clones with stable straight rod shape, the FACS sort also yielded clones with helical morphologies that have lower side curvature, possibly an indicator of a smaller helical radius, as well as clones with longer helical pitch compared with wild-type. Although the FACS-identified mutants displayed a range of morphological aberrations during logarithmic growth, they all transitioned to coccoid morphology in late stationary phase (∼ 72 h of culture in broth) similar to wild-type (data not shown).

### Csd6 is an ld-carboxypeptidase that acts upstream of Csd4 in PG modification

Both *csd6* and *mviN* have annotations suggesting they may encode proteins that alter PG. We therefore performed muropeptide analysis of PG sacculi isolated from these mutant strains. The Δ*mviN* mutant PG muropeptide profile was very similar to wild-type except for a slight decrease in dipeptide content (2.8% of total PG in WT compared with 1.7% in Δ*mviN*, a decrease of 39%) (Table 3). In contrast, the *csd6* mutants (transposon insertion or targeted deletion) had nearly undetectable levels of monomeric dipeptides and tripeptides in the sacculus as well as undetectable levels of tetra–tripeptide cross-linked species. In addition, the *csd6* mutants showed elevated levels of monomeric tetrapeptides and tetra–tetrapeptides, but not tetra–pentapeptides. These results suggest Csd6 cleaves monomeric and perhaps cross-linked tetrapeptides to produce tripeptides. The *csd6* complemented strain showed perturbations of the same species as the mutant except in the opposite direction (increases in tripeptides and decreases in tetrapeptides), suggesting that the complemented strain has enhanced Csd6 activity. In support of this conclusion, the *csd6* merodiploid strain contained even higher levels of tripeptide species and lower levels of tetrapeptide species compared with the *csd6* complemented strain.

**Table 3:** Summary of the muropeptide composition of mutant strains

Muropeptide^b^	Area – % of each muropeptide^a^										
	WT (Avg. ± SD)^c^	*mviN*	*csd6^tn^*	*csd6*	*csd6^cmpl^*	*csd6 OP*	*csd4*^c^	*csd4 OP*	*csd4csd6*	*csd1*^d^	*csd1csd6*
Monomers (total)	58.7 ± 1.7	57.3	57.4	57.5	57.8	57.6	60.0	56.7	57.0	53.3	54.0
Di	2.8 ± 0.4	1.7	**0.6**	**0.5**	2.8	4.4	**0.0**	4.0	**0.2**	2.3	**0.0**
Tri	4.0 ± 0.4	4.6	**0.0**	**0.0**	**8.4**	**11.1**	**16.1**	**1.8**	**0.0**	3.8	**0.0**
Tetra	10.0 ± 0.6	10.0	**15.9**	**16.1**	**6.2**	**3.9**	**2.1**	10.1	**15.4**	8.6	**14.1**
Penta	41.8 ± 1.1	41.0	40.9	41.0	40.4	38.2	41.8	40.8	41.4	38.6	39.9
Dimers (total)	41.3 ± 1.7	42.7	42.6	42.5	42.2	42.4	40.0	43.3	43.0	46.7	46.0
TetraTri	4.5 ± 0.3	4.5	**0.0**	**0.0**	**8.5**	**11.7**	**13.2**	3.3	**0.0**	4.0	**0.0**
TetraTetra	15.8 ± 0.3	15.8	**20.7**	**21.1**	**11.9**	**9.8**	**7.5**	16.7	**21.5**	16.8	**20.3**
TetraPenta	21.1 ± 1.3	22.4	21.9	21.3	21.8	20.9	19.3	23.3	21.5	26.0	25.7
Chain ends (with anhydro-MurNAc)	10.3 ± 0.6	10.3	10.5	10.3	10.2	10.9	9.0	10.7	10.4	10.5	10.6
Avg. chain length	9.7 ± 0.6	9.7	9.5	9.7	9.8	9.2	11.1	9.3	9.6	9.5	9.4
% Peptides in cross-links	41.3 ± 1.7	42.7	42.6	42.5	42.2	42.4	40.0	43.3	43.0	46.7	46.0

a Percentages calculated as per (Glauner, 1988). Underlined values differ by more than 2 standard deviations from that of wild-type; underlined and bold values differ by more than 5 standard deviations from that of wild-type. Strains used: LSH100, TSH1, P1S1G1_5, TSH17, TSH31, TSH35, LSH28, LSH79, TSH27, LSH154, DBH11.

b Nomenclature according to Glauner (1988).

c Wild-type and *csd4* mutant values as previously reported (Sycuro *et al.*, 2012), the former calculated from six independent samples.

d Similar to *csd1* mutant values previously reported (Sycuro *et al.*, 2010).

Prior work showed that Csd4 acts to remove the terminal *meso*-Dap residue of monomeric tripeptides, a predicted product of Csd6 activity. Moreover, genetic interaction studies using a Δ*csd1csd4* double mutant indicated Csd4 carboxypeptidase activity was independent of Csd1 mediated hydrolysis of tetra–pentapeptide cross-links in the PG sacculus (Bonis *et al.*, 2010; Sycuro *et al.*, 2010). When we investigated the muropeptide profile of a Δ*csd1csd6* double mutant, we found that it appeared additive to that of Δ*csd1* and Δ*csd6* single mutants, showing both the elevated tetra–pentapeptide dimers characteristic of Δ*csd1* mutant sacculi and the elevated tetrapeptides and reduced tri- and dipeptides characteristic of Δ*csd6* mutant sacculi (Table 3). Likewise, quantitative cell shape analysis demonstrated that the Δ*csd1csd6* double mutant has a side curvature distribution that is intermediate to and significantly different from those of both parent strains (Fig. S4A and B). In contrast, the Δ*csd4csd6* double mutant had a muropeptide profile identical to that of Δ*csd6* single mutants (suggesting Csd4 has a functional dependence on Csd6), particularly with respect to tripeptide and tetrapeptide containing species (Table 3). The Δ*csd4csd6* double mutant also has straight rod morphology similar to that of both parent strains (Fig. S4C and D). Thus, both muropeptide and cell shape analyses indicate that Csd6 and Csd4 act together in a PG peptide trimming pathway that functions independently of Csd1 cross-linking relaxation to both modify the PG sacculus and directly or indirectly generate helical cell shape. These findings further designate Csd6 as the sole peptidase in *H. pylori* that can produce the tripeptide substrate for Csd4, but proteins in addition to Csd1 (likely Csd3 and other unidentified proteins) produce the tetrapeptide substrate of Csd6.

Given this evidence that Csd6 acts upstream of Csd4, we wondered if overexpression of Csd4 would perturb cell shape in a manner similar to that observed when we overexpressed Csd6. As shown in Fig. S5A–C, a *csd4* merodiploid strain contains a significant subpopulation of straight rods not observed in the wild-type or *csd4* complemented strain. Using CellTool's proxy measure of cell length (the length of the cell centreline drawn pole to pole on the cell's two-dimensional image), we also observed a positive association between cell length and *csd4* gene dosage (Fig. S5D), where the Δ*csd4* mutant's length distribution was shorter than wild-type (*P* < 0.00001) and the *csd4* merodiploid strain was longer (*P* < 0.00001). The length distributions for the *csd6* strains or the Δ*csd4csd6* double mutant were not statistically different from that of wild-type (*P* > 0.05 for all other comparisons). Since levels of monomeric dipeptide and tripeptide are both directly (though in one case inversely) correlated with predicted Csd4 and Csd6 protein expression levels across the mutant, wild-type and merodiploid strains, these data suggest that proper levels of one or both of these monomeric peptides are critical for helical morphology. Other aspects of Csd4 function, potentially its interactions with other proteins, may be important for determining *H. pylori*'s cell length.

### Csd6 protein converts monomeric tetrapeptides in isolated sacculi to tripeptide and correlates with tripeptide content in cells

**Fig 4:**
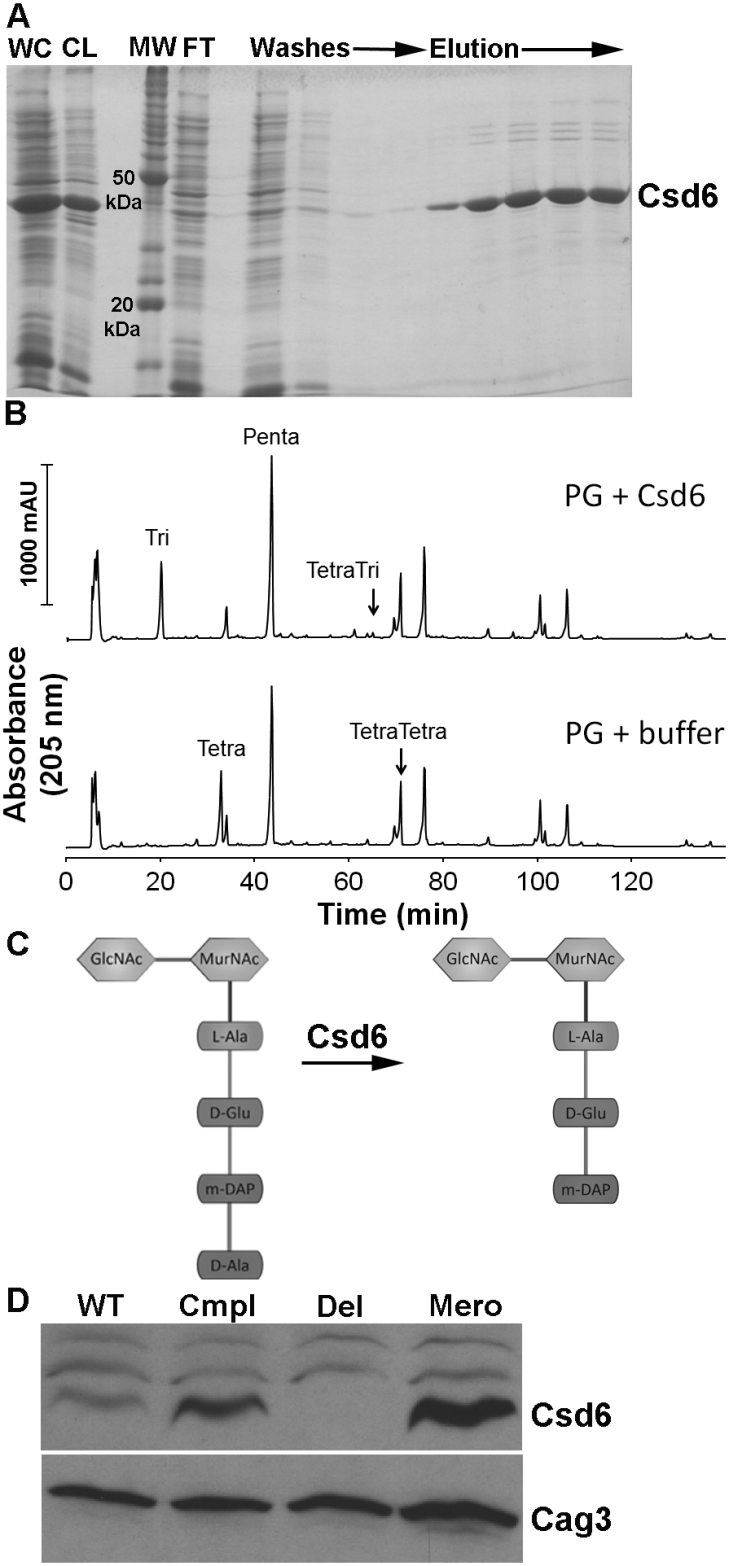
Functional analysis of Csd6 enzymatic activity and expression levels. A. SDS-PAGE gel depicting steps in the purification of oligohistidine-tagged *H. pylori* Csd6 protein from *E. coli* cells. The protein was purified using Ni-NTA resin as described in *Experimental procedures*. WC, induced whole-cell lysate; CL, cleared lysate; MW, molecular weight markers; FT, flow-through. B. HPLC analysis of muropeptides released from purified peptidoglycan (PG, obtained from the Δ*csd1csd6* mutant, strain DBH11) upon treatment with His-Csd6 or buffer followed by cellosyl digestion. Loss of monomeric tetrapeptides with formation of monomeric tripeptides in the presence of Csd6 is indicative of the protein having ld-carboxypeptidase activity. C. Schematic of the predicted activity of Csd6 based on experiment in (C) showing the substrate (tetrapeptide) and product (tripeptide). D. Antibody-based detection of Csd6 in whole-cell extracts prepared at equal cell density. Blots were stripped and re-probed with antisera against another periplasmic protein, Cag3, for quantitative expression analyses. One of three representative experiments is shown. WT, wild-type (LSH100); Cmpl, *csd6* complement (TSH31); Del, *csd6* null allele (TSH17), Mero, *csd6* merodiploid (TSH35).

To further explore the activity of Csd6, we overexpressed hexahistidine-tagged Csd6 (His-Csd6) in *E. coli* (Fig. 4A). Incubation of purified His-Csd6 with tetrapeptide-rich sacculi from the Δ*csd1csd6* mutant resulted in complete conversion of the monomeric tetrapeptides to tripeptides, while the cross-linked tetra–tetrapeptide and tetra–tripeptide showed almost no change (Fig. 4B and Table S1). Thus Csd6 is an ld-carboxypeptidase capable of cleaving monomeric tetrapeptides in the PG sacculus (Fig. 4C), although it may influence conversion of tetra–tetrapeptides to tetra–tripeptides indirectly or in concert with an unknown cofactor.

Antibodies prepared against purified Csd6 protein were used to probe whole-cell extracts of wild-type, Δ*csd6* mutant, *csd6* complement and *csd6* merodiploid strains (Fig. 4D). Quantification of the Csd6 band and that of an unrelated (control) periplasmic protein, Cag3, revealed a 2.1-fold increase in Csd6 protein expression in the complemented strain and a 2.6-fold increase in the merodiploid strain compared with wild-type. The increased protein expression levels observed for the complemented and merodiploid strains may account for the higher levels of tripeptide and lower tetrapeptide (Table 3) and the increased abundance of cells with straight rod morphology observed in these strains (Fig. 2) compared with wild-type.

## Discussion

We have demonstrated that a single round of FACS using a low FSC inclusion gate was highly successful in enriching our *H. pylori* transposon mutant pool for straight rod cell shape mutants. Although the methodology utilized in this proof-of-concept study still involved tedious microscopy-based investigation of the morphologies of individual clones recovered in the FACS output, it was clear that after sorting, clones with a morphology phenotype were no longer rare, and indeed were nearly a majority of what we observed through the microscope. Although we have not yet attempted multiple rounds of sorting, we predict this would further improve shape mutant enrichment; too many rounds, however, could reduce the genetic and morphological complexity of the pool. The efficiency of the technique also would have been even greater had we not recovered many clones with unstable alterations in cell shape. This unexpected finding is suggestive of a small subpopulation of cells in the parent *H. pylori* strain that transiently exhibit low curvature across multiple passages and periods of logarithmic growth. The reason for this morphological instability is currently unknown, but it could result from epigenetic regulation of shape-determining genes or other stochastic events occurring on a single cell level. Incidentally, we also do not fully understand the reason for the morphological heterogeneity seen in some of our genetically altered strains such as the Δ*csd3* mutant and the *csd6* complement and merodiploid strains described in this study, though in the latter case, heterogeneity seems to correlate with Csd6 protein expression and resultant tripeptide levels. Thus another potentially powerful use of the morphological sorting capability we have developed is separation of morphological subpopulations from isogenic cultures, allowing for investigations of how morphology relates to the cell's transcriptome, proteome and intrastrain variation in PG content.

Among the clones with uniform straight rod morphology obtained in our sort, most mapped to the two loci identified in our original screen, *csd4* and *csd5*. Size heterogeneity of the PCR products obtained with transposon and gene-specific primers indicated that multiple insertions within these genes were present in the sorted population. In contrast, only a single clone was identified as containing an insertion in the *csd6* locus, whose role in cell shape we describe here for the first time. This would seem to suggest that transposon insertions in *csd6* were under-represented in our library, although it is also possible that clones containing *csd6* insertions were, by chance, less abundant in the library plating. Constructing additional libraries with more random target specificity than the Tn*7*-based system used to generate this library (Salama *et al.*, 2004) and screening more densely populated library platings could therefore facilitate identification of additional shape genes.

The *csd6* gene (also called HP0518 based on the gene locus identifier in strain 26695) was previously identified in a screen for altered motility in soft agar and suggested to diminish flagellin glycosylation through an unknown mechanism (Asakura *et al.*, 2010). We previously found straight rod *csd4* mutants show altered mobility in soft agar and we are currently testing the motility of both *csd4* and *csd6* mutants in purified gastric mucin to explore shape effects on motility. A homologue of *csd6* from another helical-shaped organism, *C. jejuni pgp2*, has been recently characterized (E. Gaynor, pers. comm.). Pgp2 protein also displays ld-carboxypeptidase activity on tetrapeptide-containing muropeptides and *pgp2* mutants show straight rod morphology and attenuated motility. Thus Csd6 and Pgp2 appear to function in a shared helical cell shape generation programme conserved among Epsilonproteobacteria.

In addition to the dominant straight rod shape, close examination of the sorted clones yielded a subset with more subtle perturbations of shape including a decrease in side curvature measurements, possibly indicating a smaller helical radius, and longer wavelength measurements, suggesting a longer helical pitch. Such clones were not retained in our microscopy-based screen, which suggests that our FACS protocol may have enriched for clones with subtle morphology phenotypes, but further FACS analyses of mutants in this ‘altered helicity’ shape class is needed to confirm this possibility. The genes disrupted in these mutants are a conserved hypothetical protein, HPG27_782, and a predicted *mviN/murJ* homologue. While *mviN* has been suggested to play an essential role in the synthesis or transport of lipid linked PG precursors in *E. coli* (Inoue *et al.*, 2008; Ruiz, 2008), this gene apparently is not essential in *H. pylori* and the only alteration observed in the muropeptide composition of the *mviN* mutant's PG sacculus was a slight decrease in dipeptide levels. A *M. tuberculosis mviN* homologue was shown to positively regulate cell growth through interaction with a forkhead domain containing protein FhaA that is a negative regulator of cell growth (Gee *et al.*, 2012). Interestingly, in addition to altering cell growth, mycobacterial *mviN* and *fhaA* mutants have altered cell length and width. The observed increase in the helical pitch of *mviN* mutants thus may underlie alterations in the kinetics, processivity or localization of cell wall incorporation during cell growth. Similarly, the small increase in the fraction of cells with lower cell curvature observed for HPG27_782 mutants suggests an indirect or regulatory role in helical cell shape generation. Further biochemical and genetic experiments will be needed to address these possibilities.

Our demonstration that Csd6 has ld-carboxypeptidase activity hydrolysing monomeric tetrapeptide to produce monomeric tripeptide identifies a predicted, but as yet undescribed PG modification activity in *H. pylori*. In the absence of Csd6, neither tripeptides nor dipeptides are detected in the PG sacculus. Csd4 has dl-carboxypeptidase activity on monomeric tripeptides, producing dipeptides in the sacculus. The Δ*csd4csd6* double mutant PG phenotype suggests these two proteins act sequentially to trim monomeric tetrapeptides to tripeptides (Csd6) and then dipeptides (Csd4), which do not undergo further modification unless broken down for recycling purposes. Biochemical assays using isolated sacculi as a substrate indicated that both Csd4 (Sycuro *et al.*, 2012) and Csd6 do not have activity on cross-linked substrates, yet both affect steady-state levels of cross-linked species when mutated. Changes in cross-linked species could result from alterations in the monomeric peptides available for cross-linking during insertion of new PG strands and thus may be indirect. Consistent with this hypothesis, the total dimers and per cent cross-linking are not perturbed in these mutants even though the relative amounts of the different cross-linked species is changed.

Our previous work suggested the peptide trimming activity of Csd4 constitutes a PG modification programme that promotes cell shape independent of Csd1-mediated cross-linking relaxation (Sycuro *et al.*, 2012). Our findings in this study suggest that Csd6 is another member of the Csd4 PG trimming pathway and provide additional clues as to how peptide trimming might contribute to cell curvature. Csd6 seems to act in concert with Csd4 to produce dipeptides, while the two proteins have opposite effects on tripeptides, suggesting that some consequence of the conversion from tripeptide to dipeptide relates to the induction of cell curvature. The significance of di- and tripeptides in cell shape determination may relate to the fact that their terminal *meso-*Dap residue (absent in dipeptides) is required for the formation of PG cross-links. Experimental and modelling studies have shown that cell curvature can be generated by asymmetrically localizing cross-links in the PG sacculus (Huang *et al.*, 2008). Hence Csd4 and Csd6 may contribute to the localized alteration of cross-links through their combined activity that eliminates cross-link acceptors (tetra- and tripeptides). Precise spatial localization could explain the loss of curvature observed with both loss and increased gene dosage of Csd4 and Csd6. According to this model, loss of expression prevents the induction of curvature while extra protein expression at additional sites may break the asymmetry of cross-links and again lead to loss of curvature. Interestingly, loss of Csd5 leads to near complete loss of curvature with no change in the global muropeptide composition of the sacculus (Sycuro *et al.*, 2012), suggesting Csd5 may play a role in the asymmetric localization of Csd4 and/or Csd6. Optimization of Csd4 and Csd6 antisera for subcellular localization studies should allow us to address this model. We also observed that increased copy number of *csd4*, but not *csd6*, resulted in increased cell length and loss of *csd4* gave rise to slightly shorter cells. This may suggest a direct or indirect interaction between Csd4 and components of the cell wall synthesis or cell division machinery. Asymmetric cell wall synthesis, which has been shown to produce curved morphology in bacteria (Cabeen *et al.*, 2009), thus represents an alternative mechanism by which these proteins may promote cell curvature.

Altogether, our findings suggest FACS is readily applicable for rapidly and efficiently isolating bacterial cells with particular cell shapes from morphologically diverse populations. Characterization of our FACS selected clones is beginning to reveal a diverse spectrum of proteins that directly or perhaps indirectly impact cell morphology, allowing us to further refine our understanding of the mechanisms underlying helical cell shape generation in *H. pylori*.

## Experimental procedures

### Bacterial strains and growth

Strains used in this work are described in Table S2. *H. pylori* were grown in Brucella broth with 10% fetal bovine serum (BB10) or on Columbia agar plates containing 10% horse blood (HB) as described (Sycuro *et al.*, 2010). For resistance marker selection HB agar plates were supplemented with 15 μg ml^−1^ chloramphenicol or 25 μg ml^−1^ kanamycin.

### Flow cytometry and cell sorting

Flow cytometry was performed using an Influx Flow Cytometer (BD Biosciences) and the results analysed with FlowJo (Tree Star). For optimal detection of small cells, we improved sensitivity in the forward scatter direction by effectively increasing the magnification of the detection system, a technique that high speed cell sorters have used previously to detect relatively weak fluorescent signals. Rather than using a simple lens to collect forward scattered laser light, we employed a high numerical aperture (NA) microscope objective (Mitutoyo 20×, Edmunds Optics, Barrington, NJ), and a second lens to focus the light from the objective onto a mirrored surface. A 1 mm pinhole located on the central axis of the mirror acts as a field stop. This system effectively separates scattered light from stray laser light, allowing the detection of particles as small as 100 nm.

Cells used in sorting experiments were obtained by making a cell suspension directly from frozen stocks, diluting them 10^−5^ to 10^−6^ in Brucella broth (BD Biosciences) with 10% fetal bovine serum (Hyclone, BB10), and plating on HB plates to obtain single colonies. After incubating microaerobically for 2–3 days, colonies were collected from the plate, prepared as a liquid culture at 0.2 optical density at 600 nm (OD_600_), and shaken for approximately 3 h (one doubling). Combinations of wild-type and shape mutants were prepared at ratios of 1:1 and 1:1000 mutant : WT with a total cell density of 10^6^ cells ml^−1^. The input and sorted output were plated to plain HB plates and HB plates containing chloramphenicol. The enrichment for shape mutants was calculated as the sorted ratio divided by the starting ratio.

Library sorting was conducted on a 10 000-clone mini-Tn*7* transposon mutant library constructed as described utilizing a chloramphenicol acetyltransferase (*cat*) selectable marker (Baldwin *et al.*, 2007). The library was plated at a 1:5000 dilution from frozen stocks to obtain ∼ 2500 single colonies per plate. Approximately 25 000 colonies were gathered from 10 plates (2.5-fold coverage of the library) yielding a total of 8.1 × 10^10^ cells. A total of 5.9 × 10^8^ cells were transferred to a broth culture (∼ 0.2 OD_600_) that was shaken for 2 h to encourage the cells to start elongating and display their morphology. The number of cells did not change significantly during this incubation. Following this incubation, 10^7^ cells were transferred into the 10 ml suspension used for cell sorting. Suspensions of wild-type and *csd4* mutant cells were prepared in the same manner to calibrate the flow cytometer and determine the desired gate position on the day of the sort. A 2000-cell output was sorted into the gate from a total of ∼ 250 000 cells. The output was collected into a microcentrifuge tube containing 100 μl BB10 and plated to two plain HB plates. The actual viable output obtained was approximately 600 colonies total. These were collected en masse and frozen in BB10 containing 20% glycerol at −80°C for future study.

### Morphological and genetic analysis of the sorted population

Single colonies of the sorted population were analysed by inoculating 100 μl BB10 mini-cultures in a 96-well plate and incubating them microaerobically for 16 h. Cell morphology was observed by light microscopy at 400× using a Nikon Eclipse TS100 inverted microscope. Shape mutants were confirmed after growth in shaken liquid culture, fixation in 4% paraformaldehyde, and observation at 400–1000× using phase-contrast microscopy. Genomic DNA was prepared from the pooled FACS output as well as individual clones using the Wizard protocol (Promega). The presence of insertions in known shape loci within the pooled population was determined using shape locus and transposon specific primers. Individual clones' insertions were mapped by performing PCR using primers flanking each shape locus; a 1 kb increase in the size of amplicon indicated the presence of a transposon insertion in the locus. Known shape loci were tested sequentially in the following order: *csd4*, *csd5*, *csd2/csd1/ccmA* and *csd3*. Transposon insertions in uncharacterized loci were mapped using PCR amplification with transposon-specific primers and degenerate primers, followed by second amplification with nested primers and capillary electrophoresis sequencing at the FHCRC genomics facility (Salama *et al.*, 2004). Sequence alignments were built using Sequencher (Genecodes). All primers utilized are listed in Table S3.

### Construction of mutant and complemented strains

Targeted disruption of *csd6*, *mviN* and HPG27_782 was accomplished using PCR SOEing (Horton, 1995) with primers listed in Table S3 to create insertion/deletion alleles that replaced > 50% of the coding sequence with a CAT cassette as described (Sycuro *et al.*, 2012). The *csd6* complemented and merodiploid strains were constructed by transforming the wild-type or mutant strains with a modified Bluescript SK vector, pTS10, containing a wild-type copy of *csd6* (HPG27_477) flanked by two 200 bp segments of DNA from a previously characterized neutral locus located between HPG27_186 and HPG27_187 (Langford *et al.*, 2006). The first step in constructing pTS10 was to amplify the flanking segments using the primers indicated in Table S3 and insert them in the Bluescript vector using the SacI and KpnI sites respectively. The *aphA3* gene from *Campylobacter coli* (Wang and Taylor, 1990) was added to the vector utilizing the SalI and XhoI sites to confer kanamycin resistance. Next, 350 bp of sequence upstream of HPG27_473, the first gene in the operon containing *csd6* (Sharma *et al.*, 2010), was added immediately downstream of the 5′ intergenic fragment using the XbaI and BamHI restriction sites. Finally, *csd6*, including 50 bp upstream of the start codon and 356 bp downstream of the stop codon was cloned in between the promoter sequence and the *aphA3* kanamycin marker, with EcoRI and ClaI, to create pTS10. Capillary electrophoresis of Sanger sequencing performed in the FHCRC genomics facility was used to confirm the correct sequence of the integrated *csd6* coding sequence. The *mviN* mutation was complemented by expression at the *rdxA* locus. This was accomplished by inserting *mviN* coding sequence into pLC292, a plasmid containing *rdxA* flanking sequences, using the XbaI and SalI restriction sites (Terry *et al.*, 2005).

### Scanning electron microscopy (SEM)

*Helicobacter pylori* cells were grown in shaken BB10 liquid culture to 0.3–0.4 OD_600_, and adhered to Thermanox (Nunc) coverslips by centrifugation for 10 min at 4000 r.p.m. Cells were fixed in half strength Karnovsky fixative (2.5% glutaraldehyde, 2% paraformaldehyde in 0.1 M cacodylate buffer), washed in 0.1 M cacodylate buffer, and post-fixed with 1% osmium tetroxide in cacodylate buffer. Cells were washed again in 0.1 M cacodylate buffer and stained with 0.5% uranyl acetate. After a final wash in 0.1 M cacodylate buffer cells were dehydrated in ethanol, dried with HMDS Chemical (Electron Microscopy Services), and sputter coated with Au/Pd. Cells were visualized with a JEOL JSM 6610LV scanning electron microscope and digital images manipulated using ImageJ and Adobe Photoshop.

### Muropeptide analysis

PG was prepared from *H. pylori* cells (100–500 ODs) grown on HB plates as described (Sycuro *et al.*, 2010). Purified PG (0.5 mg ml^−1^) was incubated with oligohistidine-tagged Csd6 (5 mM) purified from *E. coli* in 20 mM sodium phosphate 100 mM NaCl, pH 4.8 for 4 h at 37°C on a thermomixer at 750 r.p.m. The samples were incubated with 10 μg of cellosyl (Hoechst, Frankfurt am Main, Germany) for 18 h, boiled for 10 min and centrifuged at room temperature for 15 min at 16 000 *g*. The muropeptides present in the supernatant were reduced with sodium borohydride and analysed by HPLC as described (Glauner, 1988; Sycuro *et al.*, 2010). Eluted muropeptides were detected by their absorbance at 205 nm. Each series of samples for PG preparation included a sample of wild-type or another appropriate control. The muropeptide profile (i.e. the retention time and relative area of all peaks) of the wild-type was very similar to the published profile of *H. pylori* muropeptides involving mass spectrometry analysis (Costa *et al.*, 1999) and to our previous analysis (Sycuro *et al.*, 2010) allowing the assignment of known muropeptide structures to the peaks detected.

### Purification of His-Csd6

The *csd6* gene, starting at bp 52 (codon 18; the first 17 amino acids of Csd6 are predicted to encode a periplasmic localization signal), was amplified by PCR (primer sequences in Table S3) and cloned into the NdeI and XhoI sites of pET15b (Novagen) to allow expression in *E. coli* of a cytoplasmic Csd6 (aa 18–303) with an N-terminal hexahistidine tag. BL21(DE3) cells carrying this plasmid were grown in LB supplemented with 100 μg ml^−1^ ampicillin at 37°C with shaking to mid-log phase (*A*_600_ 0.6), induced with 1 mM IPTG, and grown for an additional 3 h. Cells were harvested by centrifugation, resuspended in purification buffer [50 mM NaH_2_PO_4_ pH 8, 500 mM NaCl, 10% glycerol, 10 mM MgCl_2_] supplemented with 1× Complete EDTA-free Protein Inhibitor Cocktail (Roche), and stored at −20°C.

Purification of His-Csd6 followed the protocol described for His-Csd4 (Sycuro *et al.*, 2012). Briefly, cells were thawed, supplemented with 1 mg ml^−1^ lysozyme, 0.2% Triton and DNase, and then lysed by three additional freeze–thaw cycles and three sonication cycles. The soluble fraction was bound to prepared Ni-NTA agarose (Invitrogen) in batch at 4°C for 1 h with gentle mixing. Beads were washed four times with 10 volumes of wash buffer (purification buffer plus 20 mM imidazole). His-Csd6 was eluted with 10 volumes of elution buffer (purification buffer plus 250 mM imidazole). Elution fractions containing the most pure, concentrated His-Csd6 were pooled. The glycerol concentration was increased to 20% prior to storage at −20°C.

### Antibody production and immunoblotting

The purified recombinant 6HisCsd6 protein (5 mg ml^−1^) was used for antibody production in rabbits by a commercial vendor (RR Rabbitry). *H. pylori* whole-cell extracts were prepared by harvesting cells by centrifugation 2 min max speed in a microcentrifuge and suspending in 2× sodium dodecyl sulphate-polyacryamide gel electrophoresis (SDS-PAGE) sample buffer (Ausubel *et al.*, 1997) at 0.01 optical density at 600 nm. Proteins separated by SDS-PAGE were transferred onto polyvinylidene difluoride membranes (Amersham) using a semidry transfer system (I-blot; Invitrogen) according to the manufacturer's instructions. Membranes were blocked for 1 h at RT or overnight at 4°C with 3% non-fat milk–Tris-buffered saline with Tween 20 (TBS-T; 0.5 M Tris,1.5 M NaCl, pH 7.4, plus 0.05% Tween 20), followed by incubation for 1 h at RT with primary antibody at a 1:10 000 dilution in TBS-T [anti-Cag3 (Pinto-Santini and Salama, 2009) and anti-Cag6]. Three 15 min washes with TBS-T were followed by a 1 h incubation at RT with horseradish peroxidase-conjugated rabbit immunoglobulin G (Santa Cruz Biotechnology) at a 1:10 000 dilution in TBS-T. After three more TBS-T washes, antibody detection was performed with an ECL Plus immunoblotting detection kit following the manufacturer's protocol (GE Healthcare). The relative densities of the Csd6 and Cag3 bands were determined using the ImageJ software (http://rsb.info.nih.gov/ij/) and Csd6 band density was normalized to Cag3 band density to compare samples.

### Shape analysis

Phase-contrast microscopy was performed as described (Sycuro *et al.*, 2010). Quantitative analysis of phase-contrast images of bacteria to measure side curvature and central axis length were performed with the CellTool software package as described (Sycuro *et al.*, 2010). ‘Axis wavelength’, an estimate of the three-dimensional helical pitch based on the 2D shape of each cell, was calculated as follows. First, an approximately sinusoidal ‘central axis’, running down the midline of each cell, was identified as described previously (Sycuro *et al.*, 2010). Next, for each point along this central axis, the perpendicular distance from that point to a straight line passing through the end-points of the bacteria was calculated. Local distance extrema identified to find the ‘peaks’ and ‘troughs’ of this curve. Average peak-to-trough distance was calculated and doubled to provide an estimate of ‘wavelength’ more robust than that produced by direct fitting of the central axis points to true sinusoids. Statistical comparison of cell shape distributions were performed using a CellTool module that calculates a bootstrap distribution of Kolmogorov–Smirnov (KS) statistics, as described (Sycuro *et al.*, 2012).
