# Supplementary material for: Flow cytometry-based enrichment for cell shape mutants identifies multiple genes that influence Helicobacter pylori morphology
Source: Mol Microbiol. 2013 Oct 16;90(4):869–83. doi: 10.1111/mmi.12405 (PMC3844677; doi:10.1111/mmi.12405)

Flow cytometry based enrichment for cell shape mutants identifies multiple genes that influence *Helicobacter pylori* morphology
Supplementary Materials

Laura K. Sycuro[1], Chelsea S. Rule[1,2], Timothy W. Petersen[3], Timna J. Wyckoff[1,4], Tate Sessler[1], Dilip B. Nagarkar[1], Fakhra Khalid[1], Zachary Pincus[5], Jacoby Biboy[6], Waldemar Vollmer[6], and Nina R. Salama[1,2]

[1]Division of Human Biology, Fred Hutchinson Cancer Research Center, Seattle, WA, [2]Department of Microbiology, University of Washington School of Medicine, Seattle, WA, [3]BD Biosciences, Seattle, WA, [4]Division of Science and Mathematics, University of Minnesota, Morris, MN, [5]Department of Molecular, Cellular and Developmental Biology, Yale University, New Haven, CT, USA, [6]Centre for Bacterial Cell Biology, Institute for Cell and Molecular Biosciences, Newcastle University, Newcastle upon Tyne, United Kingdom.

Inventory:

Table S1. Muropeptide composition of His-Csd6 treated *csd6csd6* mutant sacculi.

Table S2. Bacterial strains

Table S3. Primers

Figure S1. Spectra illustrating the approximate position of gates utilized to enrich for shape mutants in morphological sorting feasibility experiments.

Figure S2. Low FSC gate used for FACS sort of *H. pylori* transposon mutant library.

Figure S3. Identification of insertions in known shape loci within the pooled FACS output population.

Figure S4. Morphological characterization of double mutants.

Figure S5. Loss or overexpression of *csd4* perturbs cell shape and length.

Table S1. Muropeptide composition of His-Csd6 treated *csd1csd6* mutant sacculi.

**Peak No.**	**Muropeptide**	**DBH11 + buffer**	**DBH11 + His-Csd6**
1	Tri	0.0	12.7
2	Tetra	13.0	0.0
3	Penta(Gly5)	4.5	4.6
4	Di	0.0	0.0
5	Penta	30.4	31.1
6	TetraTri	0.0	0.6
7	TetraPenta(Gly5)	3.8	3.3
8	TetraTetra	9.3	9.0
9	TetraPenta	14.1	13.5
10	PentaAnh	1.6	1.7
11	TetraTriAnh I	0.0	0.7
12	TetraTriAnh II	0.0	0.1
13	TetraTetraAnh I	6.9	6.1
14	TetraTetraAnh II	2.0	1.6
15	TetraPentaAnh	7.5	7.3
1 - 15	all known	93.0	92.5

Table S2. Bacterial strains.

Name	Relevant Genotype or Description	Reference or Source
NSH57	Wild-type *H. pylori*: mouse-adapted G27	(Baldwin et al., 2007)
LSH100	Wild-type *H.pylori*: NSH57 with *fliM* repaired	(Lowenthal *et al.*, 2009)
LSH18	Δ*csd4::catsacB* in NSH57	(Sycuro et al., 2012)
KGH10	Δ*ccmA::catsacB* in NSH57	(Sycuro et al., 2010)
LSH112	Δ*csd3::catsacB* in NSH57	(Sycuro et al., 2010)
P1S1G1_5	*csd6:tnGPScat* in NSH57	this study
TSH17	*Δcsd6::cat* in LSH100	this study
TSH31	*Δcsd6::cat McGee:csd6:aphA3* in LSH100	this study
TSH35	*McGee:csd6:aphA3* in LSH100	this study
LSH79	*rdxA::csd4* in NSH57	this study
LSH104	Δ*csd4::catsacB* rdxA::csd4 in NSH57	(Sycuro et al., 2012)
P1S1G1_6	HPG27_458:*tnGPScat* in NSH57	this study
P1S1G1_9	HPG27_866:*tnGPScat* in NSH57	this study
P1G1S1_11	HPG27_355*:tnGPScat* in NSH57	this study
P1G1S1_12	HPG27_1132:*tnGPScat* in NSH57	this study
P1S1G1_20	HPG27_782:*tnGPScat* in NSH57	this study
MHH17	ΔHPG27_782::*cat* in NSH57	this study
P4G1S1_29	*mviN*:*tnGPScat* in NSH57	this study
TSH1	Δ*mviN*::*cat* in LSH100	this study
TSH13	Δ*mviN*::*cat rdxA::mviN* in LSH100	this study
LSH154	Csd1H250A in NSH57	(Sycuro et al., 2010)
LSH28	Csd4E222A in NSH57	(Sycuro et al., 2012)
DBH11	Csd1H250A Δ*csd6*::*cat* in NSH57	this study
TSH27	Csd4E222A Δ*csd6*::*cat* in NSH57	this study
DH10B	*E. coli* cloning strain	Invitrogen
BL21(DE3)	*E. coli* protein expression strain	Invitrogen

Table S3. Primers.

Name	Sequence
Transposon primers	
N3	TTTAATACGACGGGCAATTTGCACTTCAG
N2	CAGTTTAAGACTTTATTGTC
S	TAATCCTTAAAAACTCCATTTCCACCCCT
S2	AGTTCCCAACTATTTTGTCC
Shape loci primers	
HPG27_353-359u-5-XhoI	CCCTCGAGATGATAGAAGCTTGCAAAGCG
HPG27_353-356d-3-EcoRI	ACATGCATAGCTCCATCAGG
HPG27_1197-311u-5	CACTGATTCTATGGGCGTTA
HPG27_1195-315d-3	CCTAAAGCGTCGGTATTGTA
HPG27_1483-955-5	GACGCTTTTTTGGTGCTAGAA
HPG27_1479-256-3	GCGCTAATAGGGGCAATGATG
HPG27_464-193u-5	AGAAAGGGAATATCCAACGC
HPG27_464-242d-3	TTACGCGCATAAATGGCTGG
Targeted disruption primers	
hp0823-5	CTCAAATCAGCGCGATTTTAGC
hp0823-3M	ATCCACTTTTCAATCTATATCTCTTCTTCAGCC TTTAAGCCC
hp0823-5M	CCCAGTTTGTCGCACTGATAACCCAAAAAGAT CCCAATAGCG
hp0823-3	AAAATCCACTAACGCAACCCC
HPG27_477FO	ACCGGCTCTGTTAATGG
HPG27_477RI	ATCCACTTTTCAATCTATATCACTCTAAACTAG GCTTGGATT
HPG27_477FI	CCCAGTTTGTCGCACTGATAACTTCACTCGCT ATGACGG
HPG27_477_RO	GAATTGGAGCTATAAGAGAGC
838.1	GCTCTAGAAATTGTTGCAATTCCCCACCAA
838.3	ATCCACTTTTCAATCTATATCCATAAAATCCCT AAGCTGTTGGT
838.2	ACGCGTCGACAAGATTACAAGCCATGATTTTA AA
838.4	CCCAGTTTGTCGCACTGATAAAAGGGGGCAA TTTAGCCCT
Complementation primers	
1 sacI	GCGCGAGCTCAAGGGTTTCTTTAGGGAT
3 sacI	GCGCGAGCTCCATCATTAACATCATTATCG
2 kpnI	GCGCGGTACCCTGTTCTAATGGGGTGTT
4 kpnI	GCGCGGTACCGGCATATTTTTCCCTTATAT
Kan1 SalI	GCGCGTCGACACAGAATTACTCTATGAAGCG
Kan2 XhoI	GCGCCTCGAGATCTAGGTACTAAAACAATTC
csd6p1 XbaI	GCGCTCTAGAGACTCCGTTCCAATCG
csd6p2 BamHI	GCGCGGATCCTACATCCCCTTTAAGTTGTAAG
csd6.1-EcoRI	GCGCGAATTCGAAGAAAAGAGCTTGCAAA
csd6.2-ClaI	GCGCATCGATAGGTTTGAGCGCGAA
Protein expression primers	
5′Ndecsd6	GCGCGCGCATATGAGTGATCGTTTGTTAG
3′XhoISTOPcsd6	GCGCGCGCTCGAGTTATTTTTCCATTATG

Figure S1. Spectra illustrating the approximate position of gates utilized to enrich for shape mutants in morphological sorting feasibility experiments. Color density plots of spectra for wild-type cells (top row) and each class of shape mutant (bottom row). Pink outlines illustrate inclusion gates used to sort mixed culture populations containing each respective mutant and wild-type. The number outside each gate provides the percent of each single strain population that would be expected to sort into the gate. Strains used: NSH57, LSH18, KGH10, LSH112.

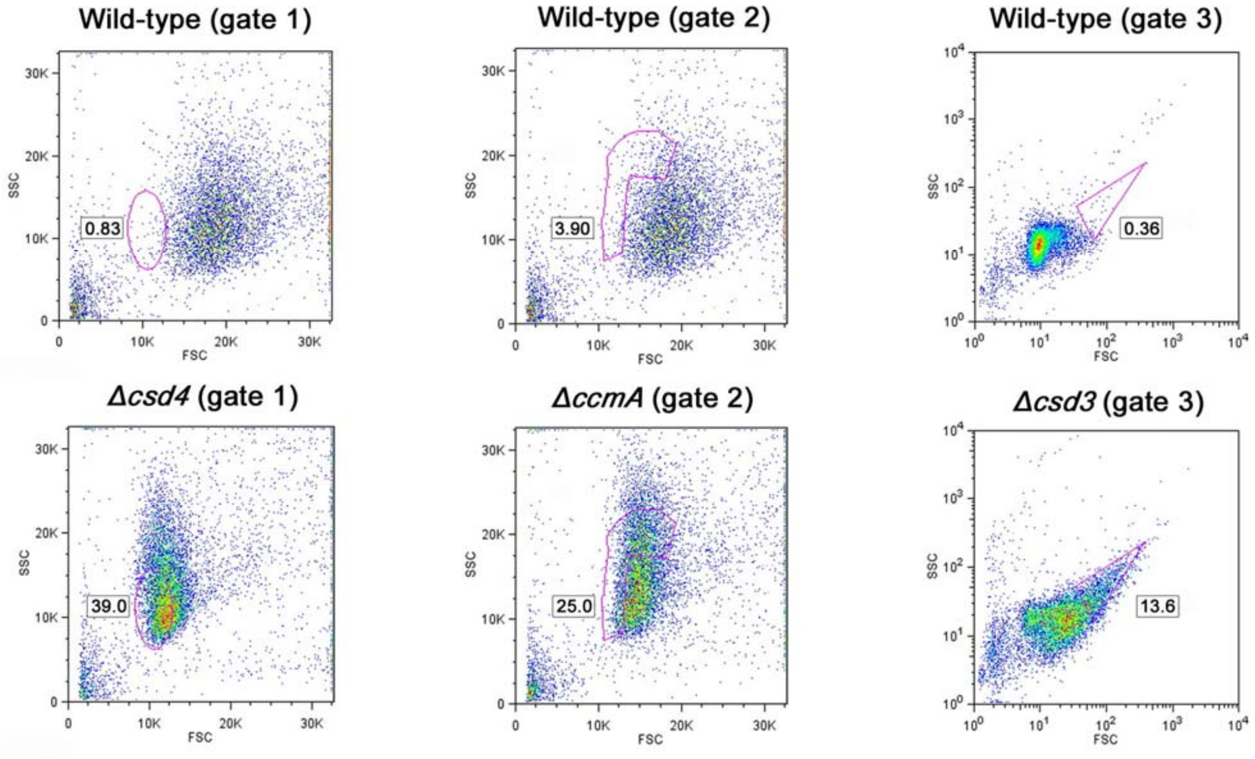

Figure S2. Low FSC gate used for FACS sort of *H. pylori* transposon mutant library. The light gray ovals show the gate position overlaying the wild-type (red arrow) and *csd4* mutant scatter plots generated on the day of the sort. ~25% the Δ*csd4* straight rod mutant and ~0.5% of the wild-type populations were included by the gate (red boxes), suggesting the library sort might be expected to enrich for straight rod mutants by 50-fold.

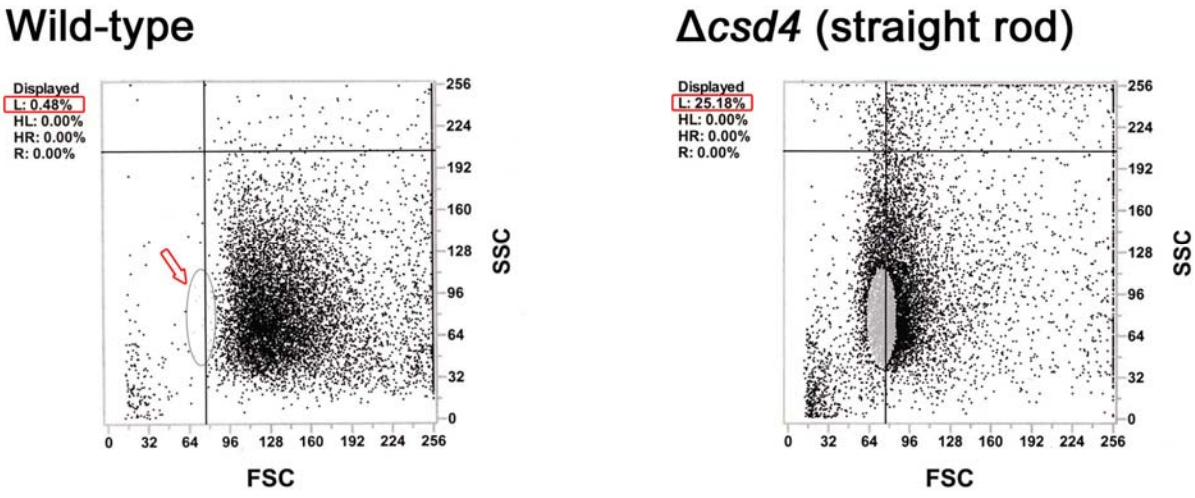

Figure S3. Identification of insertions in known shape loci within the pooled FACS output population. CEKG PCR was performed using a primer upstream (5′) or downstream (3′) of each shape locus paired with primer N3, which anneals within the transposon, as described in Experimental Procedures. M, 1 kb DNA ladder; F, forward oriented insertions detected with 3' primer; R, reverse oriented insertions detected with the 5' primer.

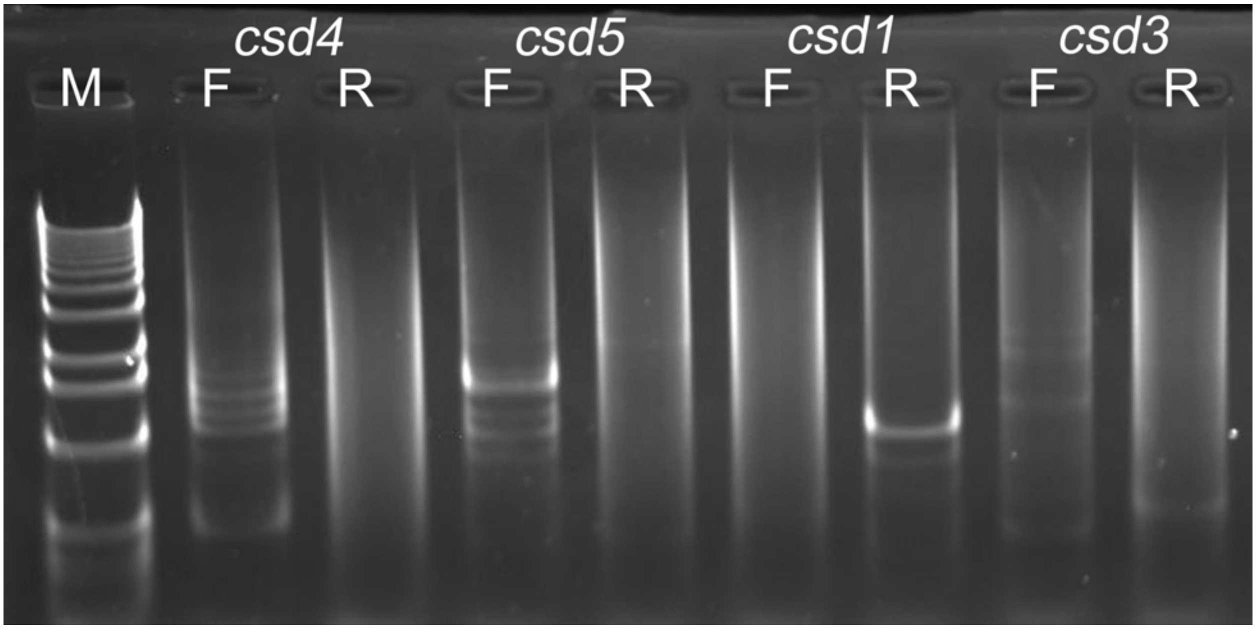

Figure S4. Morphological characterization of double mutants. A,C) Scatter plots arraying indicated populations by cell length (x-axis, μm) and cell curvature (y-axis, arbitrary units). B,D) Smooth histograms displaying population cell curvature (x-axis) as a density function (y-axis). Bootstrapped Komologorov–Smirnov (KS) statistics of population cell curvature distributions: wild-type vs. all strains $p<0.00001$, *csd1* vs. *csd6* $p<0.00001$, *csd1* vs. *csd1csd6* $p=0.00003$, *csd6* vs. *csd1csd6* $p<0.00001$, *csd4* vs. *csd6* $p=0.32304$, *csd4* vs. *csd4csd6* $p=0.00043$, *csd6* vs. *csd4csd6* $p=0.00136$. Strains used: LSH100, LSH13, LSH18, TSH17, DBH11, TSH27.

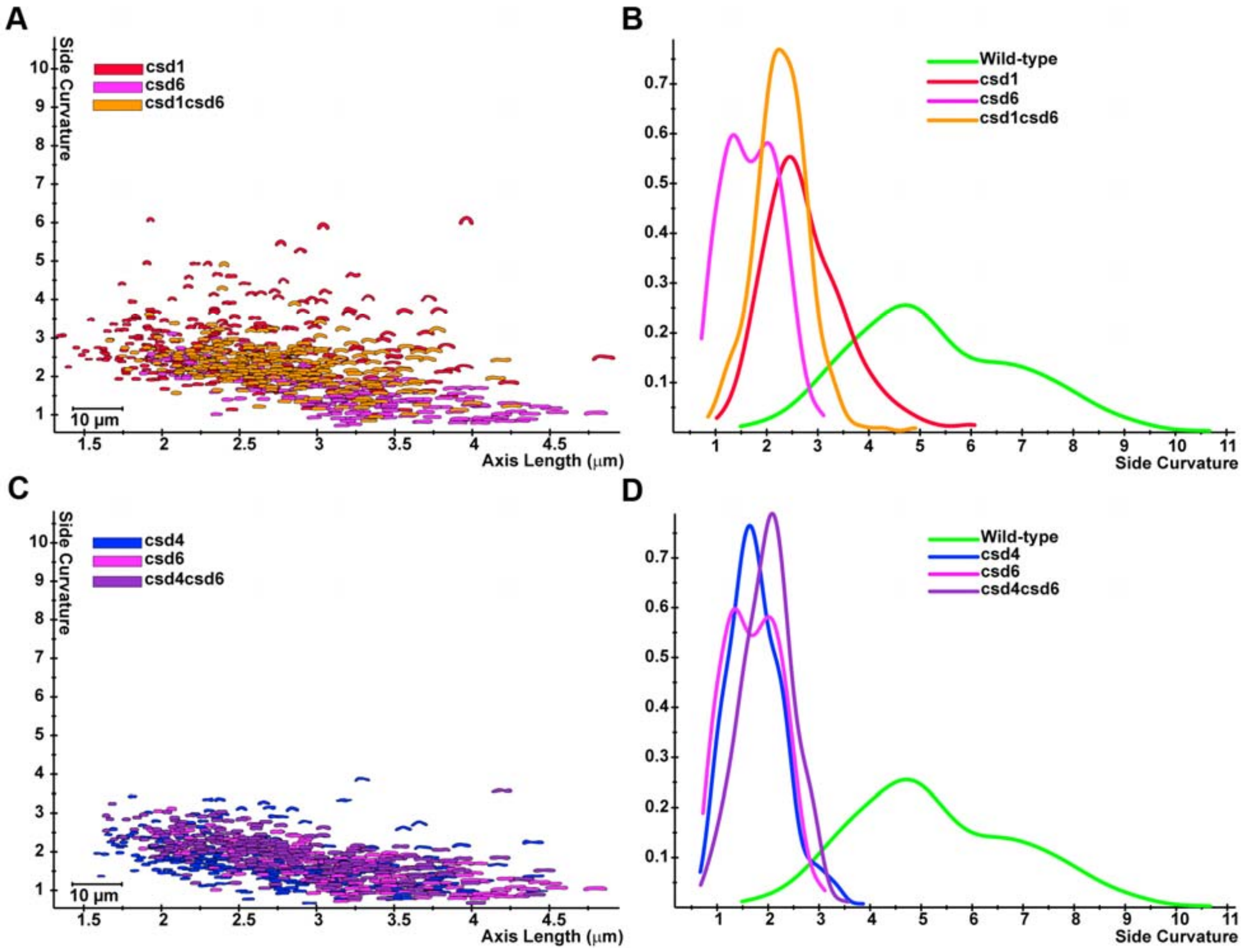

Figure S5. Loss or overexpression of *csd4* perturbs cell shape and length. A–B) Scatter plots arraying cell length (x-axis, µm) and cell curvature (y-axis, arbitrary units). Each point represents the outline of the cell image obtained by phase contrast microscopy. C) Smooth histogram displaying population cell curvature (x-axis, arbitrary units) as a density function (y-axis). D) Smooth histogram (kernel density estimate) displaying axis length (x-axis, µm) as a density function (y-axis). *csd4* mutant (Δ*csd4*), complemented (*csd4*cmpl) and merodiploid (*csd4*OP). Strains used: LSH100, LSH18, LSH104, LSH79.

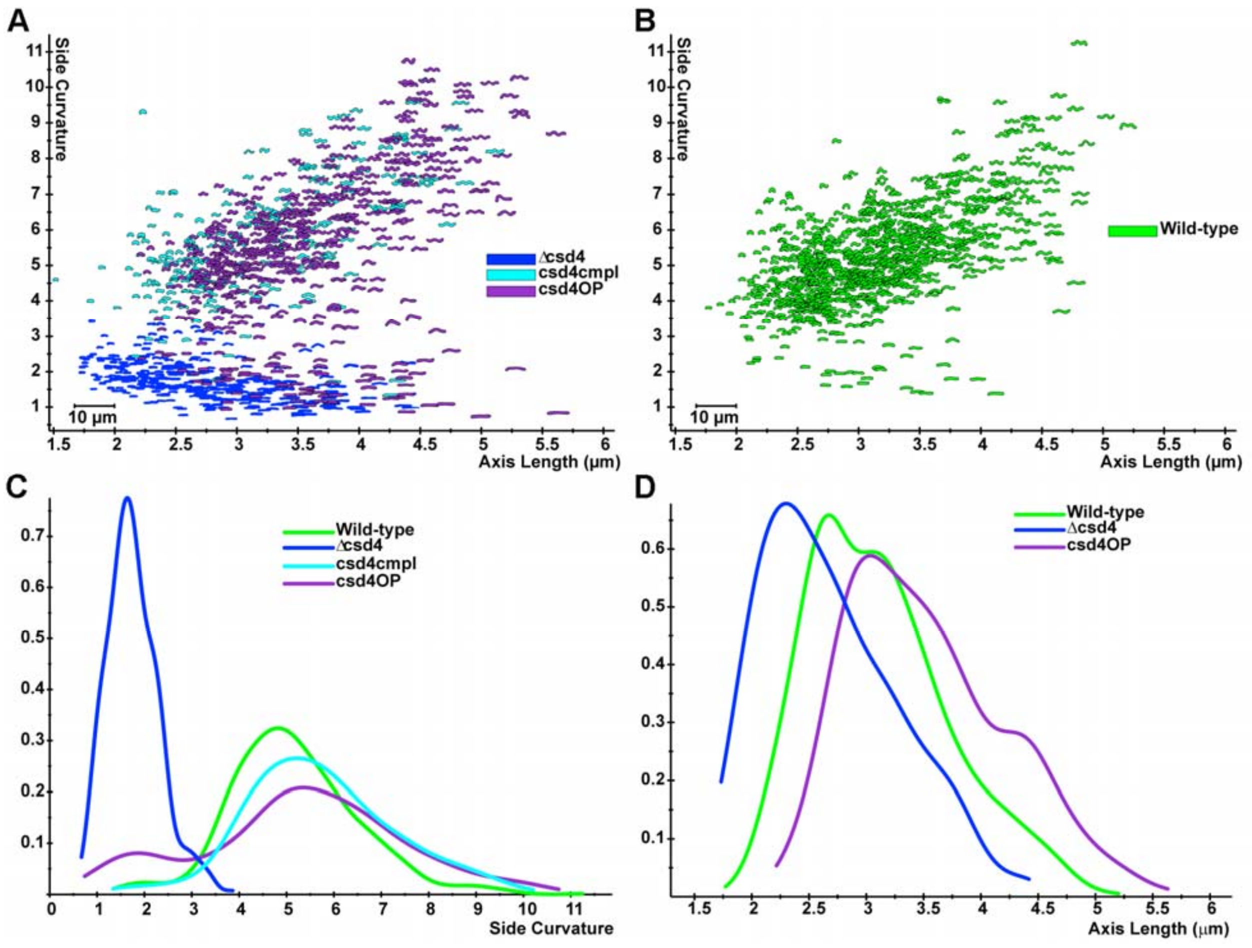

Supplement: Supplementary file 1 — Supporting Information [file mmi0090-0869-SD1.pdf]
